# Supplementary material for: Computational Investigation of Smooth Muscle Cell Plasticity in Atherosclerosis and Vascular Calcification: Insights from Differential Gene Expression Analysis of Microarray Data
Source: Bioengineering (Basel). 2025 Nov 9;12(11):1223. doi: 10.3390/bioengineering12111223 (PMC12650549; doi:10.3390/bioengineering12111223)
Supplement: Supplementary file 1 [file bioengineering-12-01223-s001.zip › TableS1_new.pdf]

Table S1. The mtry (number of variables randomly sampled as candidates at each split) parameter and overall statistics of random forest assay

Function	train	randomForest
Parameters	mtry = 11195	ntree = 1000, mtry = 12, nodesize = 1, maxnodes = 50
Accuracy	1	0.6842
95% CI	0.9501, 1	0.4345, 0.8742
No Information Rate	0.375	0.4211
P-Value [Acc > NIR]	2.2e-16	0.01874
Kappa	1	0.5128
Multi-class area under the curve	1	0.7667

CI represents confidence interval. Acc represents accuracy. NIR represents no information rate.
