# Supplementary material for: Computational Investigation of Smooth Muscle Cell Plasticity in Atherosclerosis and Vascular Calcification: Insights from Differential Gene Expression Analysis of Microarray Data
Source: Bioengineering (Basel). 2025 Nov 9;12(11):1223. doi: 10.3390/bioengineering12111223 (PMC12650549; doi:10.3390/bioengineering12111223)
Supplement: Supplementary file 1 [file bioengineering-12-01223-s001.zip › TableS2_new.pdf]

Table S2. The confusion matrix and statistics by class of random forest

	train					randomForest				
Reference Prediction	Non-SMC	SMC	SMC-ath	SMC-calc	SMC-t	Non-SMC	SMC	SMC-ath	SMC-calc	SMC-t
Non-SMC	13	0	0	0	0	1	0	0	0	0
SMC	0	27	0	0	0	2	8	2	1	1
SMC-ath	0	0	8	0	0	0	0	0	0	0
SMC-calc	0	0	0	12	0	0	0	0	2	0
SMC-t	0	0	0	0	12	0	0	0	0	2
Statistics by Class	Non-SMC	SMC	SMC-ath	SMC-calc	SMC-t	Non-SMC	SMC	SMC-ath	SMC-calc	SMC-t
Sensitivity	1.0	1.0	1.0	1.0	1.0	0.3333	1.0	1.0	0.6667	0.6667
Specificity	1.0	1.0	1.0	1.0	1.0	1.0	0.4545	1.0	1.0	1.0
Pos Pred Value	1.0	1.0	1.0	1.0	1.0	1.0	0.5714	NaN	1.0	1.0
Neg Pred Value	1.0	1.0	1.0	1.0	1.0	0.8889	1.0	0.8947	0.9412	0.9412
Prevalence	0.1806	0.375	0.1111	0.1667	0.1667	0.15789	0.4211	0.1053	0.1579	0.1579
Detection Rate	0.1806	0.375	0.1111	0.1667	0.1667	0.5263	0.4211	0.0	0.1053	0.1579
Detection Prevalence	0.1806	0.375	0.1111	0.1667	0.1667	0.05263	0.7368	0.0	0.1053	0.1053
Balanced Accuracy	1.0	1.0	1.0	1.0	1.0	0.66667	0.7273	0.5	0.8333	0.8333

NaN represents not a number
